# Associations of marital status with diabetes, hypertension, cardiovascular disease and all-cause mortality: A long term follow-up study

**DOI:** 10.1371/journal.pone.0215593

**Published:** 2019-04-22

**Authors:** Azra Ramezankhani, Fereidoun Azizi, Farzad Hadaegh

**Affiliations:** 1 Prevention of Metabolic Disorders Research Center, Research Institute for Endocrine Sciences, ShahidBeheshti University of Medical Sciences, Tehran, Iran; 2 Endocrine Research Center, Research Institute for Endocrine Sciences, ShahidBeheshti University of Medical Sciences, Tehran, Iran; International University of Health and Welfare, School of Medicine, JAPAN

## Abstract

**Background:**

To investigate the associations of marital status with major clinical outcomes including type 2 diabetes (T2D), hypertension, cardiovascular disease (CVD) and all-cause mortality.

**Methods:**

The study cohort (1999–2014) included 9,737 (45% male) Iranian adults with a mean age of 47.6 years. Marital status was defined as married versus never married, divorced and widowed. The relationship between marital status and the four above mentioned outcomes were investigated using Cox regression models adjusted for the main confounders, specific to each outcome.

**Results:**

After more than 12 years of follow-up, 1,889 (883 men) individuals developed hypertension, 1,038 (468 men) T2D, 1015 (597 men) CVD and 668 (409 men) all-cause mortality. Compared with married, being never married in men was associated with higher risk of hypertension [hazard ratio (HR): 1.55; 95% confidence interval (CI), 1.11–2.16] and all-cause mortality (2.17; 0.95–5.00; p-value = 0.066) after adjusting for confounders. Among women, compared with married status, widowed status was associated with a lower risk of T2D (0.74; 0.56–0.97) in the confounders adjusted model. Moreover, never married women had a lower risk of hypertension (0.58; 0.37–0.90) compared to married ones in the age adjusted model, a finding that did not achieve significance, after further adjustment for confounders.

**Conclusion:**

We found that the relationship between marital status and health outcomes varied by gender. Being never married was an important risk factor for hypertension and tended to be a significant risk factor for mortality in men. However, among women, being widowed was associated with a lower risk of T2D.

## Introduction

Marriage, since ancient times, has always been a fundamental social institution and plays an important role in the lives of most people[1].Over the last half-century, numerous studies from different disciplines have investigated relations between marital status and various aspects of health, including cardiovascular, immune, psychiatric and behavioral-related indices [2]. However, findings on the relationship between marital status and health or mortality have been inconsistent [3]. A number of studies conducted on samples from various ethnic groups have reported that rate of all-cause and cause-specific mortality are higher among those who are unmarried, relative to their married counterparts, a relationship which is independent of various sociodemographic characteristics [4, 5]. Recently, a meta-analysis of 34 studies with more than two million participants has demonstrated the influence of marital status on the incidence of cardiovascular disease (CVD) and the prognosis after CVD [6]. The results of this meta-analysis showed that unmarried participants had increased odds of CVDs, compared with married participants. Furthermore, a number of studies have assessed relations between marital status and other chronic illness such as hypertension [7] and type 2 diabetes (T2D) [8]. A prospective study of Atherosclerosis Risk in Communities (ARIC) data [9]found that marital status was not associated with hypertension, but among women, remaining single throughout the study period was associated with an increased risk of developing T2D.A recent study showed that not being married, and more specifically, widowhood was associated with an increased risk of T2D in men [10].On the other hand, the meanings of marriage, gender roles and family structure have changed considerably over the last few decades [11]. Mean age at first marriage has increased and many people never want to get married. People divorce and remarry several times [11] and more and more women are joining the workforce [2]. Hence, despite the large number of existing scientific studies, the impact of marital status on health outcomes still remains an interesting topic among scholars, practitioners, and general communities[12].There are limited numbers of prospective studies assessing the associations of marital status and major health outcomes in the Middle East, namely in Iran with the fundamental demographic and cultural changes over the past several decades [13].

The aim of the current study was to examine the association between each marital status (married, never married, widowed and divorced) and hypertension, diabetes, CVD and all-cause mortality in the Tehran Lipid and Glucose Study (TLGS), a large ongoing prospective cohort of Iranian population.

## Materials and methods

### Study population

**Fig 1:**
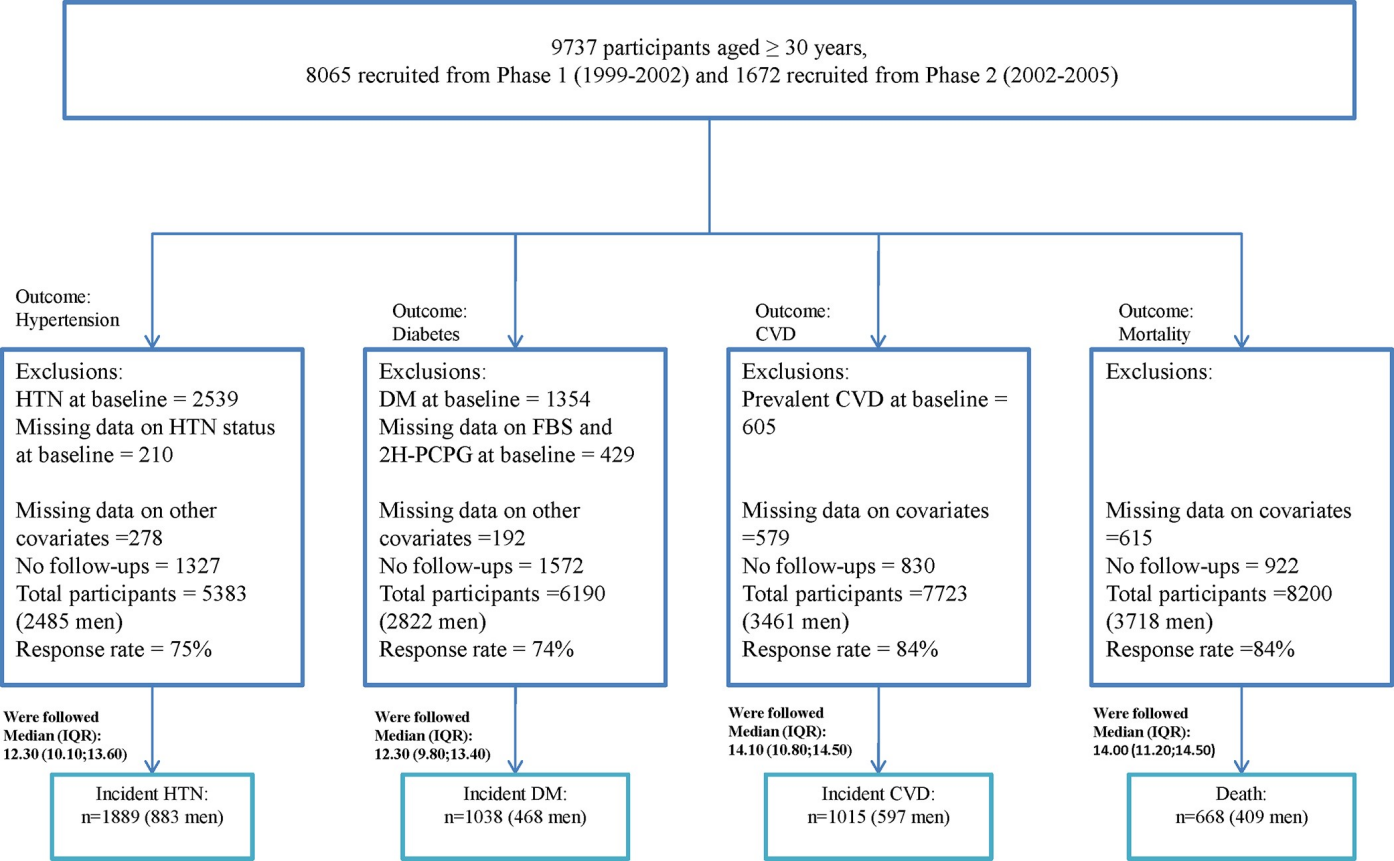
Flowchart of final analytical sample size for participants included in the study. HTN: hypertension; DM: diabetes mellitus; CVD: cardiovascular disease; IQR: interquartile range.

This research was conducted using the TLGS cohort database, an ongoing prospective study of women and men, aimed at determining the risk factors and outcomes for non-communicable disease; the design of the cohort has been described previously [14]. Briefly, a total of 15,000 and 3,551 individuals, aged ≥3 years were enrolled in the first (1999–2002) and second phases (2002–2005), respectively. For this study, we selected 9,737 participants (4,412 men), aged ≥30 years with baseline data collected in two phases (8,065 individuals from phase 1 and 1,672 participants from phase 2). Of these individuals, and for the analysis on incidence of hypertension, diabetes, CVD and all-cause mortality, we excluded those with the conditions mentioned and missing data regarding these conditions at baseline, those with missing data on covariates, and finally, those with no follow-up data after recruitment until the end of the study (20 March 2014), leaving 5,383 (2,485 men), 6,190 (2,822 men), 7,723 (3,461 men) and 8,200 (3,718 men) individuals for each analysis, respectively. Fig 1 presents a flowchart of the final analytical sample sizes, the number of participants excluded, the medians of follow up and incident cases for each outcome. The study protocol was approved by the ethics committee of the Research Institute for Endocrine Sciences of Shahid Beheshti University of Medical Sciences, Tehran, Iran, and written informed consents were obtained from all participants before inclusion.

### Data collection

Participants completed a standardized questionnaire at baseline within formation on their age, sex, marital status, smoking, medication use (antihypertensive and anti-diabetic agents) and past medical history of CVD, family history of T2D and premature CVD. Height and weight were measured based on the standard protocols, and body mass index (BMI) was calculated as weight [kg]/height[m^2^]. Systolic and diastolic blood pressure (SBP, DBP) were measured as the mean of two measurements, taken on the right arm at an interval of five minutes, with the subject resting for atleast15 minutes before the first measurement. Blood samples were collected from participants after an overnight fast of 12–14 hours to assess fasting plasma glucose (FPG), 2-h post load plasma glucose (2 h-PLPG) (measured by the enzymatic colorimetric glucose oxidase), and total cholesterol (TC) (was assayed using the enzymatic colorimetric method) [14].

### Definition of terms

A current smoker was defined as a person who smokes cigarettes or uses other tobacco products daily or occasionally. A past-smoker was a formerly daily or occasional smoker who currently does not smoke and non-smokers were defined as people who never smoked before. Family history of premature CVD was defined as any experience of chronic heart disease (CHD) or stroke, if it had occurred before 55 years of age in male relatives and before 65 years of age in female relatives. Family history of diabetes (FHD) was defined as having T2D in first-degree relatives. Hypertension was defined as a SBP ≥140 mmHg or a DBP ≥90 mmHg or taking antihypertensive medications according to guidelines of the Joint National Committee on Prevention, Detection, Evaluation, and Treatment of High Blood Pressure (JNC 7) [15]. T2D was defined as FPG ≥7 mmol/L or 2 h-PLPG ≥11.1 mmol/L [16] or using glucose-lowering treatment.

### Exposure

The primary exposure of interest was self-reported marital status. Participants listed their marital status into one of four categories: 1) married (reference category);2) never married;3) divorced; and 4) widowed. In men, due to the small numbers of participants in the widowed and divorced categories, these two groups were combined into a single group and are referred to as the widowed/divorced group.

### Outcomes

The occurrence of hypertension, T2D, CVD and all-cause mortality during the study period were considered as outcomes. CVD was defined as any CHD events plus fatal and non-fatal stroke. CHD was defined as definite myocardial infarction (MI) (diagnostic electrocardiograph and biomarkers), probable MI (positive electrocardiograph findings plus cardiac symptoms or signs plus missing biomarkers or positive electrocardiograph findings plus equivocal biomarkers), angiographic proven CHD and congestive heart failure (CHF). All-cause mortality was defined as death from any causes. The diagnosis of CVD events were eventually evaluated by a committee consisting of an endocrinologist, an internist, a cardiologist, an epidemiologist and other invited experts as needed. Details of the CVD outcome data have been previously described [17].

### Statistical methods

Baseline characteristics of the original population among marital groups were compared by ANOVA test for continuous variables were normally distributed within the groups and had the same variances in each group. The Kruskal-Wallis test was used for continuous variables with non-normal distribution in each marital group and unequal variances among groups. The Pearson Chi-square test was applied for categorical variables.

Comparison of baseline characteristics between respondents (those with complete data at baseline who had at least one follow-up data) and non-respondents (those with missing data at baseline or without any follow-up data) was performed by Student's t-test for continuous variables and the chi-square test for categorical variables.

Incidence density and mortality rates and 95% confidence interval (CI) for each outcome were calculated per 1,000 persons-years.

Cox proportional hazards (Cox PH) models were first used to assess interactions between marital status and gender in multivariate models for each outcome. As significant interactions were observed between gender and marital status in relation to hypertension incidence (p-value <0.001), all subsequent analyses were sex-stratified. Cox PH was then used to estimate hazard ratios (HRs) of developing each outcome for never married, widowed/divorced (as a single category in men), widowed and divorced individuals versus married participants. For all analysis, model 1 was the crude (unadjusted) model. Model 2 was adjusted for age (years). In Model 3, we additionally adjusted for known confounders, including BMI and smoking (for all outcomes), TC (only for hypertension analysis), T2D (except for T2D analysis), hypertension (except for hypertension analysis), FHD (only for diabetes analysis), family history of CVD (only for CVD analysis) and prevalent CVD (only for mortality analysis). The PH assumptions in the Cox models were checked using statistical tests based on the scaled Schoenfeld residuals and log-log plots.

For hypertension and T2D, the event date was defined as the mid-time between the date of the follow-up visit when the diagnosis was made for the first time, and the most recent follow-up visit prior to the diagnosis. For CVD and mortality, the event date was the exact date of events. Survival time was calculated as the time between baseline and the event date (for event cases) or the last follow-up (for censored cases). Regarding hypertension, T2D and CVD, participants were censored due to death from a cause other than these outcomes, loss to follow-up, or the end of the observation period without the event occurring. For mortality event, censoring was due to loss to follow-up and being alive at end of study (20 March 2014). All analyses were performed using the R statistical package, v.3.4.0 (www.r-project.org). Two-tailed p values<0.05 were considered significant.

## Results

The original population (n = 9,737; 45% men) included in the analysis had a mean±SD age of 47.6±12.7 years (range 30–89 years). Baseline characteristics of men and women according to their marital status have been presented in Tables 1 and 2, respectively. There were significant differences in baseline characteristics between different groups of marital status among the male population, except for DBP, smoking, FHD and family history of CVD. Among women, there were statistically significant differences for all baseline characteristics between different marital groups, except for family history of CVD.

**Table 1:** Baseline characteristics of men, by marital status;Tehran Lipid and Glucose Study (1999–2014).

	Never married n = 213	Married n = 4153	Widowed/divorced^§^n = 46	P value
**Continuous variables**				
** **Age (years)^*^	33 (5)	47 (22)	53 (35)	<0.001
** **BMI (kg/m^2^)^**†**^	24.8 (4.1)	26.2 (3.9)	25.5 (4.6)	<0.001
** **SBP (mmHg)^*^	113 (17)	119 (22)	120 (27)	<0.001
** **DBP (mmHg) ^**†**^	77.4 (9.4)	78.4 (11.3)	76.2 (11.1)	0.214
** **FPG (mmol/L)^*^	4.9 (0.5)	5.1 (0.8)	5.0 (1.5)	<0.001
** **2 h-PLPG (mmol/L)^*^	5.4 (2.1)	5.8 (2.5)	6.1 (3.0)	<0.001
** **Total cholesterol (mmol/L)^*^	4.8 (1.4)	5.3 (1.3)	5.2 (1.7)	<0.001
**Categorical variables, frequency (%)**				
** **Smoking^‡^				
** **Never	120 (56.3)	2081(50.1)	20 (43.4)	0.074
** **Past	20 (9.3	673 (16.2)	9 (19.5)	
** **Current	67 (31.4)	1293 (31.3)	16 (34.7)	
** **Diabetes mellitus^‡^				
** **No	193 (90.6)	3358 (80.8)	33 (71.7)	<0.001
** **Yes	6 (2.8)	562 (13.5)	10 (21.7)	
** **Hypertension^‡^				
** **No	179 (84.0)	3023 (72.7)	38 (82.6)	<0.001
** **Yes	24 (11.3)	1014 (24.4)	7 (15.2)	
** **Family history of diabetes				
** **No	159 (74.6)	3076 (74.1)	37 (80.4)	0.609
** **Yes	54 (25.4)	1077 (25.9)	9 (19.6)	
** **Prevalent CVD				
** **No	212 (99.5)	3837 (92.4)	42 (91.3)	<0.001
** **Yes	1 (0.5)	316 (7.6)	4 (8.7)	
** **Family history of CVD				
** **No	190 (89.2)	3550 (85.5)	43 (93.5)	0.102
** **Yes	23 (10.8)	603 (14.5)	3 (6.5)	

* Values are presented as median (interquartile range), and P value is calculated with Kruskal Wallis test.

†Values are presented as mean (SD), and P value is calculated with ANOVA test.

‡Data contain missing values when the cell percentages do not add up to 100%.

^**§**^The group included 21 widowed and 25 divorced men.

**BMI**: body mass index; **FPG**: fasting plasma glucose; **2 h-PLPG**; 2-h post load plasma glucose; **CVD**: cardiovascular disease; **SBP**: systolic blood pressure; **DBP**: diastolic blood pressure; **SD**: standard deviation

**Table 2:** Baseline characteristics of women, by marital status; Tehran Lipid and Glucose Study (1999–2014).

	Never married n = 204	Married n = 4433	Divorced n = 95	Widowed n = 593	P value
**Continuous variables, mean (SD)**					
Age (years)^*^	34 (8)	44 (17)	44 (10)	62 (10)	<0.001
BMI (kg/m^2^) ^**†**^	24.9 (4.6)	28.6 (4.7)	28.1 (4.8)	29.0 (4.8)	<0.001
SBP (mmHg)^*^	108 (14)	117 (24)	112 (16.9)	130 (22.3)	<0.001
DBP (mmHg) ^**†**^	74.3 (9.8)	78.8 (10.7)	76.7 (9.7)	80.9 (11.5)	<0.001
FPG (mmol/L)^*^	4.8 (0.5)	5.0 (0.8)	4.9 (0.7)	5.2 (1.4)	<0.001
2 h-PLPG (mmol/L)^*^	5.4 (1.8)	6.2 (2.3)	5.6 (2.4)	6.8 (4.0)	<0.001
Total cholesterol (mmol/L)^*^	4.9 (1.4)	5.5 (1.5)	5.5 (1.1)	6.1 (1.4)	<0.001
**Categorical variables, frequency (%)**					
** **Smoking^**‡**^					
** **Never	194 (95.1)	4042(91.1)	75 (79.7)	518 (88.8)	<0.001
** **Past	3 (1.5)	93 (2.1)	8 (8.5)	39 (6.6)	
** **Current	6 (2.9)	227 (5.1)	11 (11.4)	26 (4.4)	
** **Diabetes mellitus^**‡**^					
** **No	188 (92.1)	3708 (83.6)	76 (86.3)	398 (69.9)	<0.001
** **Yes	8 (3.9)	585 (13.1)	12 (13.6)	171(30.0)	
** **Hypertension^**‡**^					
** **No	190 (93.1)	3200 (72.1)	77 (81.1)	281 (47.4)	<0.001
** **Yes	12 (5.8)	1163 (26.2)	18 (18.9)	301 (50.8)	
** **Family history of diabetes					
** **No	128 (62.7)	3077 (69.4)	55 (57.9)	388 (65.4)	0.006
** **Yes	76 (37.2)	1356 (30.5)	40 (42.1)	205 (34.6)	
** **Prevalent CVD					
** **No	203 (99.5)	4229 (95.4)	91 (95.8)	518 (87.4)	<0.001
** **Yes	1 (0.5)	204 (4.6)	4 (4.2)	75 (12.6)	
** **Family history of CVD					
** **No	173 (84.8)	3617 (81.6)	75 (78.9)	478 (80.6)	0.528
** **Yes	31 (15.2)	816 (18.4)	20 (21.1)	115 (19.4)	

* Values are presented as median (interquartile range), and P value is calculated with Kruskal Wallis test.

†Values are presented as mean (SD), and P value is calculated with ANOVA test.

‡Data contain missing values when the cell percentages do not add up to 100%.

**BMI:** body mass index; **FPG:** fasting plasma glucose; **2 h-PLPG**; 2-h post load plasma glucose; **CVD**: cardiovascular disease; **SBP**: systolic blood pressure; **DBP**: diastolic blood pressure; **SD**: standard deviation

The comparisons between non-respondents and respondents for each outcome are shown in S1–S4 Tables. Generally, compared with non-respondents, respondents were younger, and had lower prevalence of current smoking (hypertension, mortality and CVD datasets), T2D (hypertension dataset), hypertension (T2D dataset) and prevalent CVD (mortality dataset). However, they had higher prevalence of T2D and hypertension in mortality and CVD datasets.

The incident cases and median (interquartile range) of follow-ups for each outcome are presented in Fig 1.The incidence/mortality rates(95% CI) per 1000 person-years were 36.1 (34.0–38.4), 16.0 (14.8–17.4), 11.0 (10.4–11.7) and 9.0 (8.2–9.9) for hypertension, T2D, CVD and mortality, respectively.

Tables 3 and 4 show unadjusted and adjusted HRs (95% CIs) for four outcomes by marital status. Table 3 shows that never married med had significantly lower risk of T2D (HR 0.52; 95% CIs 0.30–0.91), CVD (0.20; 0.09–0.44) and all-cause mortality (0.29; 0.13–0.66). After adjusting for age, never married men had significant increased risk for hypertension (1.41; 1.01–1.97), compared to married men in age adjusted model. After further adjustment for potential confounders, risk of hypertension increased slightly (1.55; 1.11–2.16) and risk of all-cause mortality reached a marginally significant level (2.17; 0.95–5.00, P value = 0.066) in the never married compared to married men.

**Table 3:** Unadjusted and adjusted hazard ratios for incidence of four major health outcomes across categories of marital status in men; Tehran Lipid and Glucose Study (1999–2014).

	Married	Never married	Widowed/divorced
**Incident diabetes (n = 2822)**			
** **N/events	2658/451	140/13	24/4
** **Unadjusted HR (95% CI)	1(Reference)	0.52 (0.30–0.91)*	1.17 (0.43–3.14)
** **Age adjusted HR (95% CI)	1(Reference)	0.75 (0.43–1.32)	1.03 (0.38–2.7)
** **Multivariate adjusted HR (95% CI)	1(Reference)	0.85 (0.48–1.49)	1.09 (0.40–2.93)
**Incident hypertension (n = 2485)**			
** **N/events	2336/838	125/39	24/6
** **Unadjusted HR (95% CI)	1(Reference)	0.84 (0.61–0.17)	0.71(0.32–1.60)
** **Age adjusted HR (95%CI)	1(Reference)	1.41 (1.01–1.97)*	0.65 (0.29–1.46)
** **Multivariate adjusted HR (95%CI)	1(Reference)	1.55 (1.11–2.16)**	0.63 (0.28–1.42)
**CVD (n = 3461)**			
** **N/events	3250/584	178/7	33/6
** **Unadjusted HR (95% CI)	1(Reference)	0.20 (0.09–0.44)***	1.03 (0.46–2.30)
** **Age adjusted HR (95%CI)	1(Reference)	0.55 (0.26–1.17)	0.69 (0.31–1.55)
** **Multivariate adjusted HR (95%CI)	1(Reference)	0.65 (0.30–1.38)	0.66 (0.29–1.48)
**All-cause mortality (n = 3718)**			
** **N/events	3503/397	179/6	36/6
** **Unadjusted HR (95% CI)	1(Reference)	0.29 (0.13–0.66)**	1.50 (0.67–3.36)
** **Age adjusted HR (95%CI)	1(Reference)	2.06 (0.90–4.73)	0.75 (0.33–1.69)
** **Multivariate adjusted HR (95%CI)	1(Reference)	2.17 (0.95–5.00)^†^	0.67 (0.29–1.52)

HR: hazard ratio; CVD: cardiovascular disease; CI: confidence interval

*p<0.05

**p<0.01

***p<0.001

†p = 0.066

**Table 4:** Unadjusted and adjusted hazard ratios for incidence of four major health outcomes across categories of marital status in women; Tehran Lipid and Glucose Study (1999–2014).

	Married	Never married	Divorced	Widowed
**Incident diabetes (n = 3368)**				
N/events	2894/496	139/12	55/9	280/53
Unadjusted HR (95% CI)	1(Reference)	0.48 (0.27–0.85)*	1.12 (0.58–2.17)	1.23 (0.92–1.63)
Age adjusted HR (95% CI)	1(Reference)	0.68 (0.38–1.21)	1.23 (0.63–2.39)	0.70 (0.51–0.94)*
Multivariate adjusted HR (95% CI)	1(Reference)	0.85 (0.47–1.52)	1.15 (0.59–2.23)	0.69 (0.51–0.93)**
**Incident hypertension (n = 2898)**				
N/events	2508/867	136/21	52/16	202/102
Unadjusted HR (95% CI)	1(Reference)	0.40 (0.26–0.62)***	1.04 (0.63–1.71)***	1.92 (1.56–2.35)***
Age adjusted HR (95% CI)	1(Reference)	0.58 (0.37–0.90)*	1.14 (0.69–1.87)	0.95 (0.76–1.18)
Multivariate adjusted HR (95% CI)	1(Reference)	0.69 (0.44–1.07)	1.11 (0.68–1.83)	0.97 (0.78–1.21)
**CVD (n = 4262)**				
N/events	3571/319	176/2	74/6	441/91
Unadjusted HR (95% CI)	1(Reference)	0.12(0.03–0.50)**	1.01 (0.45–2.28)	2.71 (2.15–3.43)***
Age adjusted HR (95% CI)	1(Reference)	0.27 (0.06–1.11)	0.96 (0.43–2.16)	0.91 (0.70–1.17)
Multivariate adjusted HR (95% CI)	1(Reference)	0.32 (0.07–1.30)	0.80 (0.35–1.80)	0.92 (0.71–1.19)
**All-cause mortality (n = 4482)**				
N/events	3733/167	177/2	76/3	496/87
Unadjusted HR (95% CI)	1(Reference)	0.25(0.06–1.03)†	0.99 (0.31–3.11)	4.45 (3.43–5.76)***
Age adjusted HR (95% CI)	1(Reference)	0.84 (0.20–3.43)	0.94 (0.30–2.95)	1.17 (0.88–1.56)
Multivariate adjusted HR (95% CI)	1(Reference)	0.92 (0.22–3.76)	0.77 (0.24–2.43)	1.14 (0.86–1.52)

**HR**: hazard ratio; **CVD**: cardiovascular disease; **CI**: confidence interval

*p<0.05

**p = 0.051

***p<0.001

†p = 0.056

As shown in Table 4, compared to married women, never married women had significantly lower risk of T2D (0.48; 0.27–0.85), hypertension (0.40; 0.26–0.62), CVD (0.12; 0.03–0.50) and all-cause mortality (0.25; 0.06–1.03) (marginally significant). Also, the risk of hypertension was higher in divorced women than married ones (1.04; 0.63–1.71). Widowed women had significantly increased risk of hypertension (1.92; 1.56–2.35), CVD (2.71; 2.15–3.43) and all-cause mortality (4.45; 3.43–5.76) than their married counterparts. In the age adjusted models, risk of hypertension remained significantly lower in never married vs. married women (0.58; 0.37–0.90); however, the risks of hypertension did not reach a level of significance in divorced and widowed women. Among widowed women, risk of T2D substantially decreased compared to married women, after adjusting for age (0.70; 0.51–0.94); all associations mentioned above were attenuated after full adjustment, except for widowed women, who showed a lower risk of T2D (0.69; 0.51–0.93) than married women.

## Discussion

To the best of our knowledge, this is the first comprehensive study to evaluate the association between marital status and major health outcomes and mortality in Iran based on a longitudinal study. In this population-based study, we found that being single in men was associated with 55% increased risk of hypertension after adjusting for traditional risk factors such as age, BMI, smoking, TC and T2D.Furthermore, we found that relative to married men, those men in the never married group had a 2.17 times highe rall-cause mortality risk (marginally significant).Among women, widowed status was significantly associated with a 31% lower risk ofT2D after adjusting for the age, BMI, smoking, hypertension and FHD.

A number of cross sectional studies have reported an independent association between marital status and hypertension. In particular, divorced/separated/widowed and never married individuals were found to have higher prevalence of hypertension, compared to their married counterparts [7]. Longitudinal studies, however, have documented somewhat inconsistent results; in a prospective study, conducted on African American population, no associations were found between marital status and change in marital status with hypertension [9]. Another prospective study in Portugal found no predictive role of marital status in the incidence of hypertension [18].A multicentre randomized controlled study examined the association of marital status with nocturnal dipping and night-time SBP among 459 adults on a controlled diet, and reported married participants had a greater likelihood of nocturnal dipping compared with unmarried counterparts. Also, married individuals had a lower nighttime SBP, which was particularly strong in men [19].

The mechanisms underlying the effect of marital status on hypertension are not entirely understood. Previous studies have suggested some explanations for the effects of marital status including psychopathological factors, neuroendocrine pathways, health behaviors (physical activity, diet, adherence), biological mediators and immune pathways [12]. It has been suggested that married men have better sleep, less stress, better moods and have a more healthy diet compared with never-married men [19].

In our study, at baseline, never married men were younger and had a lower mean of BMI, compared with married men. In the crude model, they had lower risk of hypertension (HR 0.84) than married men, although, this association did not quite reach a statistically significant level. In the age adjusted model, they exhibited a 41% increased risk for incidence of hypertension, compared to married men. When we replaced age with other confounders (BMI, smoking, TC and T2D), the risks remained lower in the never married, compared with married men (data not shown). This highlights the role of age as an important confounder in this relationship.

In contrast to men, we founda42% lower risk of hypertension for never-married women, compared to married women in the age adjusted model. When adjusted with additional confounders including age, BMI, smoking, TC and T2D, never-married women were still at a lower risk for developing hypertension, although the association did not achieve significance.

In our study, the gender differences in the relation between marital status and hypertension incidence may be attributed to specific gender norms and values in Iran that limit women for risky behaviors such as drug use and alcohol drinking [20], which are the major risk factors of hypertension [21, 22]. Current studies show a growing rate of high-risk behaviors among Iranian men compared to women [20, 23].

Compared to married men, unmarried men, including never married and divorced/widowed, in some studies have been shown to have an increased risk of mortality. In a prospective study of 7,000 middle aged British men, never married men had a higher risk of CVD mortality (HR:1.5), compared with married men [24]. In another study conducted on 13,889 Scottish men and women (mean age 52.3 years), the confounder adjusted risk of CVD mortality was higher in never married men and widowed/divorced women, compared with their married counterparts [25]. A Chinese study showed that marriage was associated with decreased risk for all-cause and CVD mortality, in both men and women [26]. In a large study of 94,062 Japanese men and women, aged 40–79 years, it was reported that never-married men and women had higher risks of all-cause mortality, compared to their married counterparts [27]. A meta-analysis of 53 studies showed that in elderly persons, marriage or support from the spouse was associated with a 5 to 15% reduction in all-cause mortality risk [5]. Two primary theories have been proposed to explain inequalities in mortality by marital status: health selection and social causation [28]. Health selection proposes a smaller likelihood of first marriage for physically and emotionally unhealthy individuals [29]. According to the social causation, marriage has a health protective effect such as reducing stress and anxieties and promoting positive healthy behaviors, while being unmarried could have adverse health effects [29, 28]. In some cases, evidence for both theories has been documented [30].

In our study, never married men had lower risk of all-cause mortality (HR 0.29) than married men in the crude model; however, they exhibited increased risk of mortality, compared to married men in the age adjusted model (HR 2.06). When the crude model was adjusted for other confounders (BMI, smoking, T2D, hypertension and prevalent CVD) in separate models, the risk of death remained significantly lower in never married compared to married men (data not shown). These findings confirm the confounding role of age in this association. Although our finding regarding the higher risk of mortality in never married vs. married men is in line with most previous findings in this gender [5, 31],due to small number of mortality events in the never married group, the results are less reliable and larger samples should be studied to obtain unbiased estimations.

We did not find inequalities in mortality rate by marital status among women, a finding which is inconsistent with some previously reported results [5, 31]; the reason for this gender-based discrepancy in our study may be attributed to the characteristics associated with psychological status. It has been shown that single men are more likely to experience loneliness than their females counterparts [31]. Also, current literature on this issue suggest that women are 1) more likely than men to provide social control to their spouses, 2) less likely than men to engage in most negative health behaviors, and 3) have more positive impact on their partners for modification of lifestyle risk factors [32, 31]. Hence, marriage and social control efforts from a spouse may reduce male mortality more than female mortality.

In contrast to previous studies, we found no significant mortality risk indivorced,widowed and married individuals. A meta-analysis of 12 studies in elderly subjects reported an 11% higher risk of mortality in widowed versus married persons [5]. Another meta-analysis of 32 prospective studies involving more than 6.5 million people from 11 different countries found a 23% higher risk of early death from all-causes among separated/divorce adults, compared to their married counterparts. Men and younger adults (aged 65 years) had significantly increased risk for early death, following marital separation/divorce than did women and older adults [33]; one reason for this discrepancy between our finding and previous research findings may be attributable to methodological issues; we combined widowed and divorced men into a single category, and therefore it was impossible to assess the relationships in each group separately. Moreover, due to cultural, ethical and social considerations in Iran, as a religious society, data on marital status was obtained in four legal categories and there was no option for couples living together without being formally married. Recent studies have reported that the prevalence of premarital relationships is rising among Iranians [34–36]. Thus, some unmarried (never married, divorced and widowed) subjects might prefer not to discuss about their sexual experiences[36]. Most previous studies have also combined the divorced and separated groups into a single category; however, in our study the item “separation” was not available to respondents. Some spouses may live separate lives under the same roof [37].

The association between T2D and marital status has been studied in a large number of cross sectional studies [38, 39]; however, there are only very limited longitudinal studies on this issue. A prospective study using the ARIC data showed that remaining single throughout the study period was associated with 34% higher risk for T2D in women [9]. A Chinese prospective study, conducted on 41,378 men, found that not being married, and more specifically, widowhood was associated with an increased risk of T2D [10].

Several mechanisms have been proposed to explain how marital status is associated with T2D including social cognitive, mental health outcomes, health behaviors, and biological mediators, e.g., allostatic processes involving cardiovascular, neuroendocrine and immune systems [8].

Interestingly and contrary to our expectations, we found a decreased risk of T2D among widowed women, compared to married ones, a risk that persisted after adjustment for potential confounders including age, BMI, smoking, hypertension and FHD, suggesting that other factors may play a role in the association observed between being widowed and T2D incidence among women. As married women often devote themselves to caring for their husband in later life, therefore, they may suffer from the effects of the burden associated with this care. Hence, women are less likely to feel stressed and more likely to feel relief after divorce or the death of a spouse [40], aspects which may help to explain the lower risk of T2D seen among the widowed women in our study, indicating the need for further research to ascertain the real mechanism of such associations.

Research studies investigating association between marital status and risk of CVD events have documented inconsistent results [41, 42]. A recently published meta-analysis of 34 prospective cohort studies with more than two million participants showed that compared with married individuals, unmarried individuals (never married, divorced/widowed) were 1.4 and 1.16 times more likely to develop CVD and CHD, respectively. Being divorced was associated with increased odds of CHD in both genders; however, widowers were prone to strokes[6].

Inconsistent with previous studies [6], we found no association between marital status and CVD risk in men and women. A possible explanation for the lack of association between CVD risk and marital status may be that other measures of socio-economic and behavioral risk factors or genetic and biological factors contribute to the development of CVD in our population. In a recently published study, we showed that modifiable risk factors such as diabetes, hypertension and current smoking account for over 70% risk for both CVD and mortality events [43]. A recent meta-analysis has shown that loneliness and social isolation are risk factors for CVD events [44]. Although it is commonly thought that marriage can insulate individuals from the ravages of loneliness, some couples may have feelings of loneliness within their marriages and vice versa, being single may increase the social connections of peoples [37].

Strengths of this study include panel data from a representative sample of Tehranian adults with the long follow-up and annually screenings to identify the occurrence of different events. However, there are several limitations that should be acknowledged; first, we had no information on all health related behaviours and socio-economic status which may have contributed to development of our study outcomes. Second, we did not have information about the number of times the participants had been married and the time of marital status (marriage, divorce, and widowhood) as well as duration of each marital status. Furthermore, like many other studies in this field [26, 45], we relied on self-reports to measure marital status which may have been biased by the tendency of respondents to answer consistent with expected social norms of their country, as is inherent to any self-reported measures. Third, due to the small number of divorced and widowed cases among men, we were unable to stratified results by these conditions. Fourth, as we mentioned in the results, we might have underestimated incidence of hypertension and T2D, given the better health status of respondents compared with non-respondents. However, in the CVD and mortality datasets, there was no consistent difference in distribution of risk factors between the two respondent and non-respondent groups; hence, the incidence of events might not be significantly affected. Fifth, as inherent in any prospective study, the exposure and confounder variables may change over time and thus, risk for development of the outcomes may alter. Lastly, this is a population-based cohort conducted on Iranians population and thus, results may not be generalizable to other populations.

## Conclusions

In this large Iranian cohort of adults, single status (never-married) was an important risk factor for hypertension and tended to be a significant risk factor for all-cause mortality among men. However, there was a significant lower risk of diabetes for widowed women compared to married women. Being married did not appear to affect the risk of developing CVD in both genders. In order to improve hypertension and mortality rates in Iranian population, it is necessary to make not only families but also healthcare professionals aware that unmarried men are at higher risk for hypertension and mortality.

## Supporting information

S1 Table Baseline characteristics of respondents and non-respondents for analyzing hypertension incidents; Tehran Lipid and Glucose study (TLGS) (1999–2014). Mean (SD) are shown for continuous variables and P value is calculated with t-test; frequency (%) are shown for categorical variables with P value based on chi-square test.
^**a**^ Data contain missing values when the cell percentages do not add up to 100%.
**BMI**: body mass index; **FPG**: fasting plasma glucose; **2 h-PLPG**; 2-h post load plasma glucose; **SBP**: systolic blood pressure; **DBP**: diastolic blood pressure; **SD**: standard deviation.
(DOCX)

S2 Table Baseline characteristics of respondents and non-respondents for analyzing type 2 diabetes incidents; Tehran Lipid and Glucose study (TLGS) (1999–2014). Mean (SD) are shown for continuous variables and P value is calculated with t-test; frequency (%) are shown for categorical variables with P value based on chi-square test.
^**a**^ Data contain missing values when the cell percentages do not add up to 100%.
**BMI**: body mass index; **FPG**: fasting plasma glucose; **2 h-PLPG**; 2-h post load plasma glucose; **SBP**: systolic blood pressure; **DBP**: diastolic blood pressure; **SD**: standard deviation.
(DOCX)

S3 Table Baseline characteristics of respondents and non-respondents for analyzing cardiovascular diseases incidents; Tehran Lipid and Glucose study (TLGS) (1999–2014). Mean (SD) are shown for continuous variables and P value is calculated with t-test; frequency (%) are shown for categorical variables with P value based on chi-square test.
^**a**^ Data contain missing values when the cell percentages do not sum up to 100%.
**BMI**: body mass index; **FPG**: fasting plasma glucose; **2 h-PLPG**; 2-h post load plasma glucose; **SBP**: systolic blood pressure; **DBP**: diastolic blood pressure; **SD**: standard deviation.
(DOCX)

S4 Table Baseline characteristics of respondents and non-respondents for analyzing all-cause mortality events; Tehran Lipid and Glucose study (TLGS) (1999–2014). Mean (SD) are shown for continuous variables and P value is calculated with t-test; frequency (%) are shown for categorical variables with P value based on chi-square test.
^**a**^ Data contain missing values when the cell percentages do not add up to 100%.
**BMI:** body mass index; **FPG:** fasting plasma glucose; **2 h-PLPG**; 2-h post load plasma glucose; **SBP:** systolic blood pressure; **DBP:** diastolic blood pressure; **SD:** standard deviation.
(DOCX)
