# Supplementary material for: Associations of marital status with diabetes, hypertension, cardiovascular disease and all-cause mortality: A long term follow-up study
Source: PLoS One. 2019 Apr 22;14(4):e0215593. doi: 10.1371/journal.pone.0215593 (PMC6476533; doi:10.1371/journal.pone.0215593)
Supplement: S1 Table — Mean (SD) are shown for continuous variables and P value is calculated with t-test; frequency (%) are shown for categorical variables with P value based on chi-square test. a Data contain missing values when the cell percentages do not add up to 100%. BMI: body mass index; FPG: fasting plasma glucose; 2 h-PLPG; 2-h post load plasma glucose; SBP: systolic blood pressure; DBP: diastolic blood pressure; SD: standard deviation. (DOCX) [file pone.0215593.s001.docx]

**S1 Table. Baseline characteristics of respondents and non-respondents for analyzing hypertension incidents; Tehran Lipid and Glucose study (TLGS) (1999-2014)**

|  | Non-respondent  **n=1815** | Respondent  **n=5383** | **P value** |
| --- | --- | --- | --- |
| **Continuous variables** |  |  |  |
| Age (years) | 45.4 (12.8) | 44.4 (11.1) | 0.003 |
| BMI (kg/m^2^) | 26.5 (4.6) | 26.9 (4.2) | 0.003 |
| SBP (mmHg) | 112.8 (12.0) | 113.4 (11.5) | 0.085 |
| DBP (mmHg) | 74.4 (8.1) | 74.8 (7.8) | 0.077 |
| FPG (mmol/L) | 5.5 (2.0) | 5.3 (1.6) | 0.005 |
| 2 h-PLPG (mmol/L) | 6.3 (2.6) | 6.3 (2.7) | 0.542 |
| Total cholesterol (mmol/L) | 5.3 (1.1) | 5.4 (1.1) | 0.062 |
| **Categorical variables, frequency (%)** |  |  |  |
| Sex |  |  |  |
| Male | 882 (48.6) | 2485 (46.2) | 0.039 |
| Female | 933 (51.4) | 2898 (53.8) |  |
| Marital status |  |  |  |
| Never married | 120 (6.6) | 261 (4.8) | <0.001 |
| Married | 1565 (86.2) | 4844 (90.0) |  |
| Widowed/divorced | 130 (7.2) | 278 (5.2) |  |
| Smoking ^a^ |  |  |  |
| Never | 1098 (60.5) | 3959 (73.5) | <0.001 |
| Past | 119 (6.6) | 430 (8.0) |  |
| Current | 412 (22.7) | 994 (18.5) |  |
| Diabetes mellitus ^a^ |  |  |  |
| No | 1304 (71.8) | 4879 (90.6) | <0.001 |
| Yes | 173 (9.5) | 504 (9.4) |  |

Mean (SD) are shown for continuous variables and P value is calculated with t-test; frequency (%) are shown for categorical variables with P value based on chi-square test.

**^a^** Data contain missing values when the cell percentages do not add up to 100%.

**BMI**: body mass index; **FPG**: fasting plasma glucose; **2 h-PLPG**; 2-h post load plasma glucose; **SBP**: systolic blood pressure; **DBP**: diastolic blood pressure; **SD**: standard deviation
