# Supplementary material for: Associations of marital status with diabetes, hypertension, cardiovascular disease and all-cause mortality: A long term follow-up study
Source: PLoS One. 2019 Apr 22;14(4):e0215593. doi: 10.1371/journal.pone.0215593 (PMC6476533; doi:10.1371/journal.pone.0215593)
Supplement: S2 Table — Mean (SD) are shown for continuous variables and P value is calculated with t-test; frequency (%) are shown for categorical variables with P value based on chi-square test. a Data contain missing values when the cell percentages do not add up to 100%. BMI: body mass index; FPG: fasting plasma glucose; 2 h-PLPG; 2-h post load plasma glucose; SBP: systolic blood pressure; DBP: diastolic blood pressure; SD: standard deviation. (DOCX) [file pone.0215593.s002.docx]

**S2 Table. Baseline characteristics of respondents and non-respondents for analyzing type 2 diabetes incidents; Tehran Lipid and Glucose study (TLGS) (1999-2014)**

|  | Non-respondent  **n=2193** | Respondent  **n=6190** | **P value** |
| --- | --- | --- | --- |
| **Continuous variables** |  |  |  |
| Age (years) | 47.5 (13.8) | 45.8 (11.8) | <0.001 |
| BMI (kg/m^2^) | 27.1 (4.9) | 27.2 (4.4) | 0.284 |
| SBP (mmHg) | 120.5 (119.3) | 119.3 (18.1) | 0.013 |
| DBP (mmHg) | 78.0 (11.1) | 78.0 (10.7) | 0.785 |
| FPG (mmol/L) | 5.1 (0.6) | 5.0 (0.5) | 0.029 |
| 2 h-PLPG (mmol/L) | 6.1 (1.7) | 6.0 (1.6) | 0.181 |
| **Categorical variables, frequency (%)** |  |  |  |
| Sex |  |  |  |
| Male | 1012 (46.1) | 2822 (45.6) | 0.335 |
| Female | 1181 (53.9) | 3368 (54.4) |  |
| Marital status |  |  |  |
| Never married | 124 (5.7) | 279 (4.5) | <0.001 |
| Married | 1887 (86.0) | 5552 (89.7) |  |
| Widowed/divorced | 182 (8.3) | 359 (5.8) |  |
| Smoking ^a^ |  |  |  |
| Never | 1394 (63.6) | 4667 (75.4) | <0.001 |
| Past | 173 (7.9) | 509 (8.2) |  |
| Current | 457 (20.8) | 1014 (16.4) |  |
| Hypertension ^a^ |  |  |  |
| No | 1505 (68.6) | 4828 (78.0) | <0.001 |
| Yes | 500 (22.8) | 1362 (22.0) |  |
| Family history of diabetes |  |  |  |
| No | 1651 (75.3) | 4554 (73.6) | 0.061 |
| Yes | 542 (24.7) | 1636 (26.4) |  |

Mean (SD) are shown for continuous variables and P value is calculated with t-test; frequency (%) are shown for categorical variables with P value based on chi-square test.

**^a^** Data contain missing values when the cell percentages do not add up to 100%.

**BMI**: body mass index; **FPG**: fasting plasma glucose; **2 h-PLPG**; 2-h post load plasma glucose; **SBP**: systolic blood pressure; **DBP**: diastolic blood pressure; **SD**: standard deviation
