# Supplementary material for: Associations of marital status with diabetes, hypertension, cardiovascular disease and all-cause mortality: A long term follow-up study
Source: PLoS One. 2019 Apr 22;14(4):e0215593. doi: 10.1371/journal.pone.0215593 (PMC6476533; doi:10.1371/journal.pone.0215593)
Supplement: S3 Table — Mean (SD) are shown for continuous variables and P value is calculated with t-test; frequency (%) are shown for categorical variables with P value based on chi-square test. a Data contain missing values when the cell percentages do not sum up to 100%. BMI: body mass index; FPG: fasting plasma glucose; 2 h-PLPG; 2-h post load plasma glucose; SBP: systolic blood pressure; DBP: diastolic blood pressure; SD: standard deviation. (DOCX) [file pone.0215593.s003.docx]

**S3 Table. Baseline characteristics of respondents and non-respondents for analyzing cardiovascular diseases incidents; Tehran Lipid and Glucose study (TLGS) (1999-2014)**

|  | Non-respondent  **n=1409** | Respondent  **n=7723** | **P value** |
| --- | --- | --- | --- |
| **Continuous variables** |  |  |  |
| Age (years) | 47.2 (13.3) | 46.8 (12.2) | 0.221 |
| BMI (kg/m^2^) | 27.2 (4.6) | 27.4 (4.5) | 0.130 |
| SBP (mmHg) | 120.9 (19.8) | 120.9 (19.5) | 0.965 |
| DBP (mmHg) | 78.4 (10.6) | 78.5 (10.9) | 0.831 |
| FPG (mmol/L) | 5.6 (2.2) | 5.5 (1.9) | 0.056 |
| 2 h-PLPG (mmol/L) | 6.6 (2.9) | 6.7 (3.1) | 0.441 |
| **Categorical variables, frequency (%)** |  |  |  |
| Sex |  |  |  |
| Male | 630 (44.7) | 3461 (44.8) | 0.484 |
| Female | 779 (55.3) | 4262 (55.2) |  |
| Marital status |  |  |  |
| Never married | 61 (4.3) | 354 (4.6) | 0.884 |
| Married | 1245 (88.4) | 6821 (88.3) |  |
| Widowed/divorced | 103 (7.3) | 548 (7.1) |  |
| Smoking ^a^ |  |  |  |
| Never | 867 (61.5) | 5792 (75.0) | <0.001 |
| Past | 86 (6.1) | 638 (8.3) |  |
| Current | 262 (18.6) | 1293 (16.7) |  |
| Diabetes mellitus ^a^ |  |  |  |
| No | 857 (60.8) | 6722 (87.0) | <0.001 |
| Yes | 156 (11.1) | 1001 (13.0) |  |
| Hypertension ^a^ |  |  |  |
| No | 909 (64.5) | 5842 (75.6) | <0.001 |
| Yes | 291 (20.7) | 1881 (24.4) |  |

Mean (SD) are shown for continuous variables and P value is calculated with t-test; frequency (%) are shown for categorical variables with P value based on chi-square test.

**^a^** Data contain missing values when the cell percentages do not sum up to 100%.

**BMI**: body mass index; **FPG**: fasting plasma glucose; **2 h-PLPG**; 2-h post load plasma glucose; **SBP**: systolic blood pressure; **DBP**: diastolic blood pressure; **SD**: standard deviation
