# Supplementary material for: Associations of marital status with diabetes, hypertension, cardiovascular disease and all-cause mortality: A long term follow-up study
Source: PLoS One. 2019 Apr 22;14(4):e0215593. doi: 10.1371/journal.pone.0215593 (PMC6476533; doi:10.1371/journal.pone.0215593)
Supplement: S4 Table — Mean (SD) are shown for continuous variables and P value is calculated with t-test; frequency (%) are shown for categorical variables with P value based on chi-square test. a Data contain missing values when the cell percentages do not add up to 100%. BMI: body mass index; FPG: fasting plasma glucose; 2 h-PLPG; 2-h post load plasma glucose; SBP: systolic blood pressure; DBP: diastolic blood pressure; SD: standard deviation. (DOCX) [file pone.0215593.s004.docx]

**S4 Table. Baseline characteristics of respondents and non-respondents for analyzing all-cause mortality events; Tehran Lipid and Glucose study (TLGS) (1999-2014)**

|  | Non-respondent  **n=1537** | Respondent  **n=8200** | **P value** |
| --- | --- | --- | --- |
| **Continuous variables** |  |  |  |
| Age (years) | 48.5 (13.8) | 47.5 (12.4) | 0.007 |
| BMI (kg/m^2^) | 27.2 (4.7) | 27.5 (4.5) | 0.125 |
| SBP (mmHg) | 122.5 (21.2) | 121.6 (19.9) | 0.148 |
| DBP (mmHg) | 78.7 (11.0) | 78.6 (11.0) | 0.609 |
| FPG (mmol/L) | 5.7 (2.3) | 5.6 (1.9) | 0.014 |
| 2 h-PLPG (mmol/L) | 6.7 (3.1) | 6.8 (3.2) | 0.654 |
| **Categorical variables, frequency (%)** |  |  |  |
| Sex |  |  |  |
| Male | 694 (45.2) | 3718 (45.3) | 0.457 |
| Female | 843 (54.8) | 4482 (54.7) |  |
| Marital status |  |  |  |
| Never married | 61 (4.0) | 356 (4.3) | 0.473 |
| Married | 1350 (87.8) | 7236 (88.2) |  |
| Widowed/divorced | 126 (8.2) | 608 (7.4) |  |
| Smoking^*^ |  |  |  |
| Never | 951 (61.9) | 6099 (74.4) | <0.001 |
| Past | 113 (7.4) | 732 (8.9) |  |
| Current | 277 (18.0) | 1369 (16.7) |  |
| Diabetes mellitus ^a^ |  |  |  |
| No | 918 (59.7) | 7036 (85.8) | <0.001 |
| Yes | 190 (12.4) | 1164 (14.2) |  |
| Hypertension ^a^ |  |  |  |
| No | 958 (62.3) | 6030 (73.5) | <0.001 |
| Yes | 369 (24.0) | 2170 (26.5) |  |
| Prevalent CVD |  |  |  |
| No | 1408 (91.6) | 7724 (94.2) | <0.001 |
| Yes | 129 (8.4) | 476 (5.8) |  |
| Family history of CVD |  |  |  |
| No | 1287 (83.7) | 6839 (83.4) | 0.390 |
| Yes | 250 (16.3) | 1361 (16.6) |  |

Mean (SD) are shown for continuous variables and P value is calculated with t-test; frequency (%) are shown for categorical variables with P value based on chi-square test.

**^a^** Data contain missing values when the cell percentages do not add up to 100%.

**BMI**: body mass index; **FPG**: fasting plasma glucose; **2 h-PLPG**; 2-h post load plasma glucose; **SBP**: systolic blood pressure; **DBP**: diastolic blood pressure; **SD**: standard deviation
